# Supplementary material for: Prokaryote communities along a source-to-estuary river continuum in the Brazilian Atlantic Forest
Source: PeerJ. 2024 Aug 13;12:e17900. doi: 10.7717/peerj.17900 (PMC11328836; doi:10.7717/peerj.17900)
Supplement: Supplemental Information 3 — Contains supplemental figures, tables, and file descriptions. [file peerj-12-17900-s003.pdf]

Supplemental Materials

Supplemental Figures

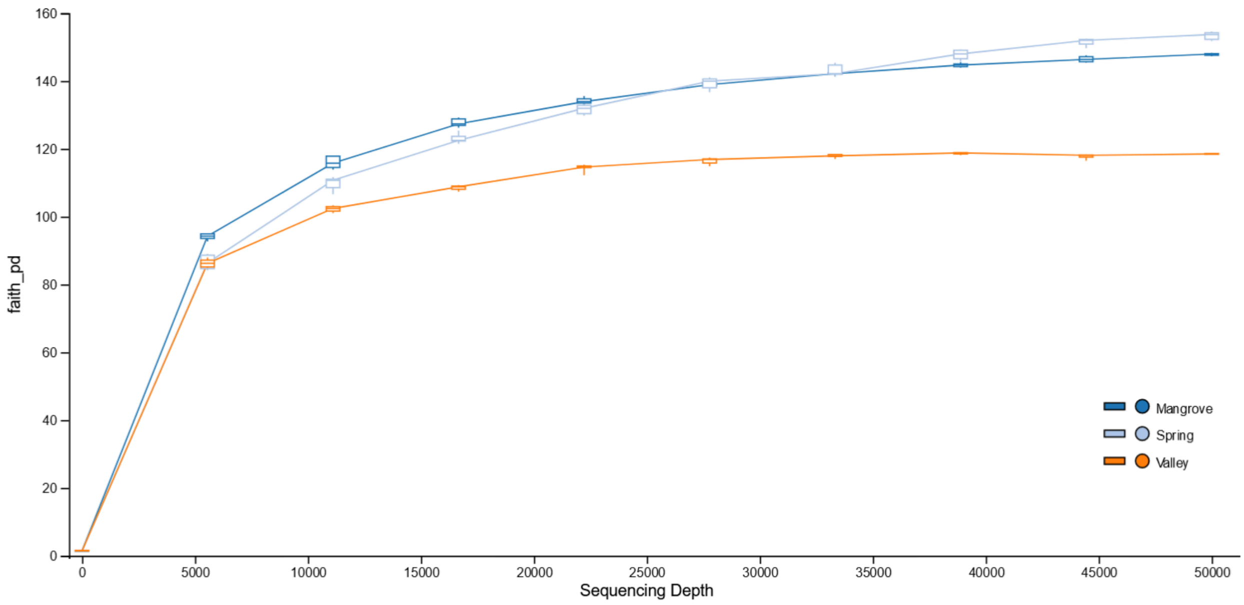

Supplemental Figure 1. Faith PD alpha-diversity rarefaction curve for each site. Based on the saturation of each site we set the rarefaction to 41K for the purposes of the alpha- and beta-diversity statistics.

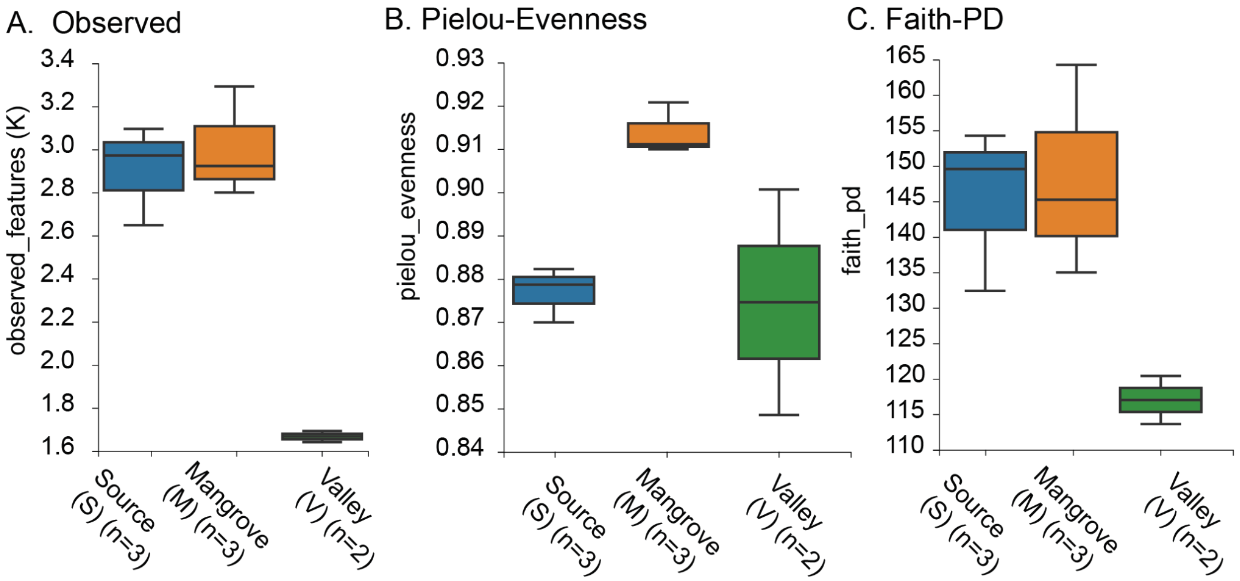

A. Observed

Kruskal-Wallis	(Omnibus)
H	4.03
p-value	0.13

Kruskal-Wallis (Pairwise)

	H	p-val	q-val
M_S	0.05	0.827	0.827
M_V	3.00	0.083	0.125
S_V	3.00	0.083	0.125

B. Pielou-Evenness

Kruskal-Wallis	(Omnibus)
H	5
p-value	0.08

Kruskal-Wallis (Pairwise)

	H	p-val	q-val
M_S	3.88	0.050	0.125
M_V	3.00	0.083	0.125
S_V	0.00	1.000	1.000

C. Faith-PD

Kruskal-Wallis	(Omnibus)
H	4.03
p-value	0.13

Kruskal-Wallis (Pairwise)

	H	p-val	q-val
M_S	0.05	0.827	0.827
M_V	3.00	0.083	0.125
S_V	3.00	0.083	0.125

Supplemental Figure 2. Alpha-diversity tests.

All alpha-diversity tests were conducted using QIIME2 on rarefied samples (p-max-depth = 41000). All tests of significance used Kruskal-Wallis.

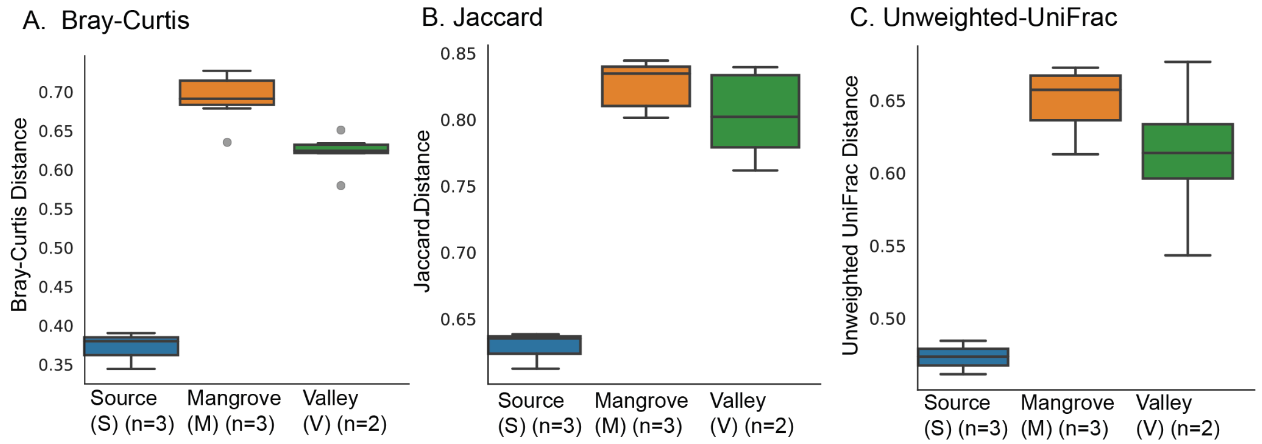

A. Bray-Curtis

PERMANOVA	(Omnibus)	
pseudo-F	4.09	
p-value	0.003	

PERMANOVA	(Pairwise)		
	pF	p-val	q-val
M_S	6.43	0.099	0.109
M_V	2.47	0.086	0.109
S_V	3.45	0.109	0.109

B. Jaccard

PERMANOVA	(Omnibus)	
pseudo-F	2.20	
p-value	0.003	

PERMANOVA	(Pairwise)		
	pF	p-val	q-val
M_S	2.92	0.097	0.097
M_V	1.60	0.096	0.097
S_V	2.01	0.094	0.097

C. Unweighted-UniFrac

PERMANOVA	(Omnibus)	
pseudo-F	2.40	
p-value	0.004	

PERMANOVA	(Pairwise)		
	pF	p-val	q-val
M_S	3.19	0.093	0.104
M_V	1.71	0.094	0.104
S_V	2.23	0.104	0.104

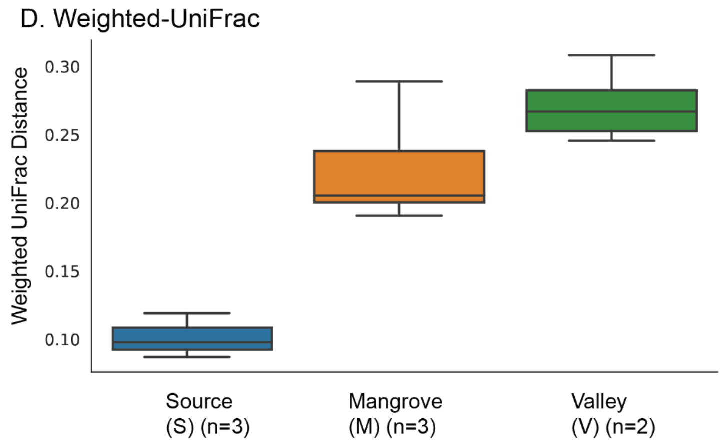

PERMANOVA	(Omnibus)	
pseudo-F	5.53	
p-value	0.007	

PERMANOVA	(Pairwise)		
	pF	p-val	q-val
M_S	6.58	0.100	0.108
M_V	4.65	0.108	0.108
S_V	5.63	0.108	0.108

Supplemental Figure 3. Beta-diversity tests.
All beta-diversity tests were conducted using QIIME2 on rarefied samples (p-max-depth = 41000). All tests used PERMANOVA on eight samples in three groups and were permuted 999 times. All figures show the distances between sites relative to the Source.

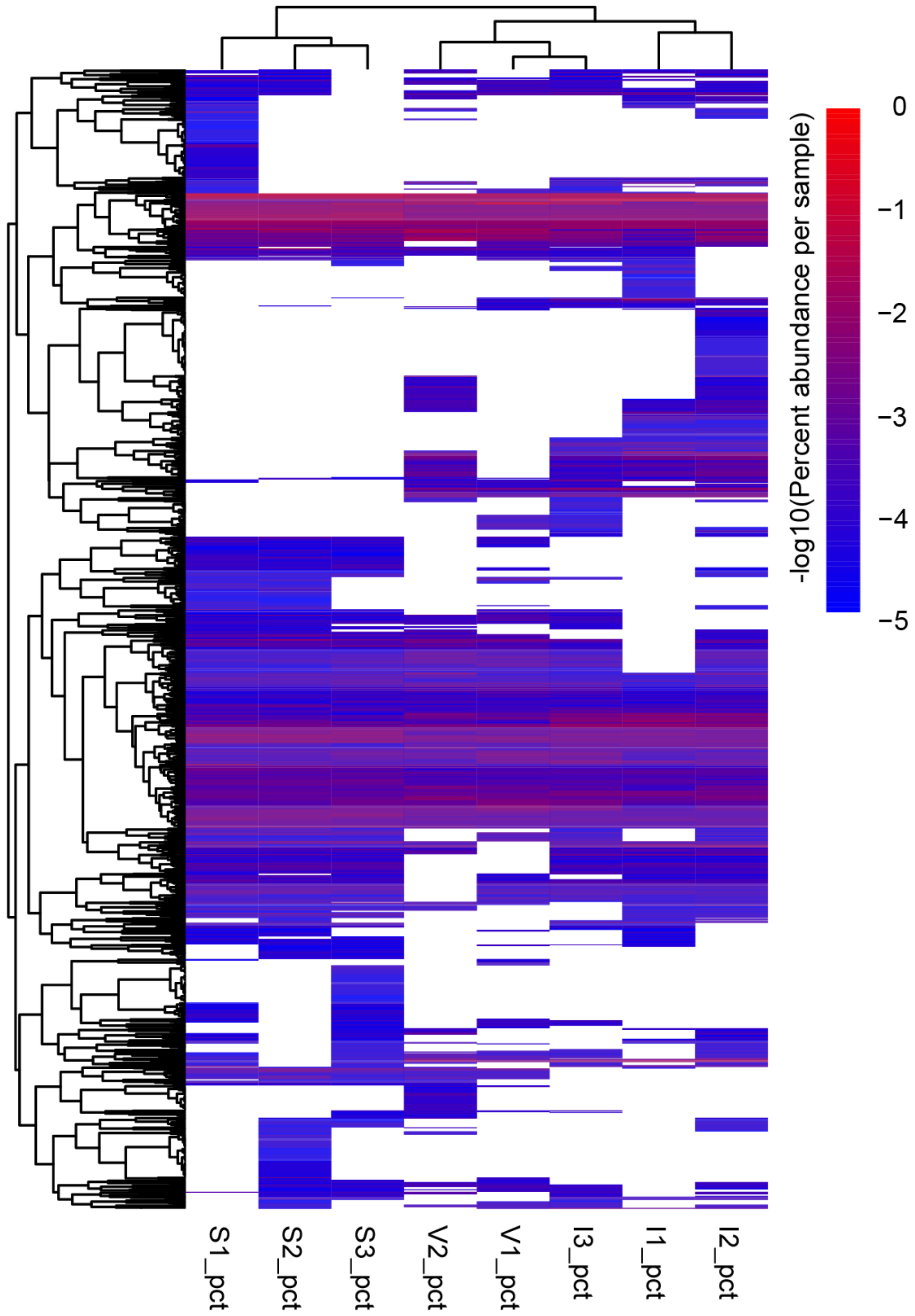

Supplemental Figure 4. Taxa abundance heatmap.
To better visualize the taxonomic diversity of the sites we represent relative abundance for each sample. Percent abundance is calculated per sample, then log10 transformed for visualization purposes. Higher abundance taxa are shown in red, lower abundance in blue. Not all taxa are present in all replicates, absence is denoted by white.

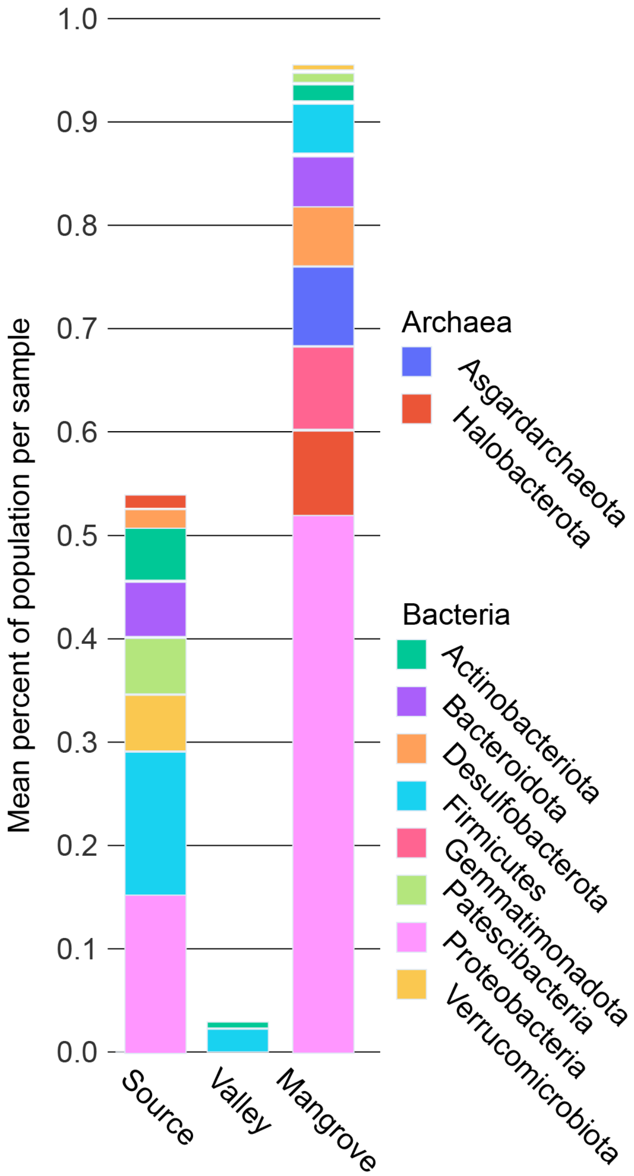

Supplemental Figure 5. Singular genera.

Figure shows the phylum level sums of genera that were present in only one site. Note that these singular genera are less than 1% of the total population at each site. The highest relative abundance genus is the Gammaproteobacteria FGL7S (0.076% of total population in Mangrove), followed by the Gammaproteobacteria Thiobacillus (0.041%, Mangrove). The highest relative abundance taxa is the Gammaproteobacteria order Chromatiales was also identified as being only present in the Mangrove site (0.109%). A complete table is available as a supplemental (Supplemental Table 8).

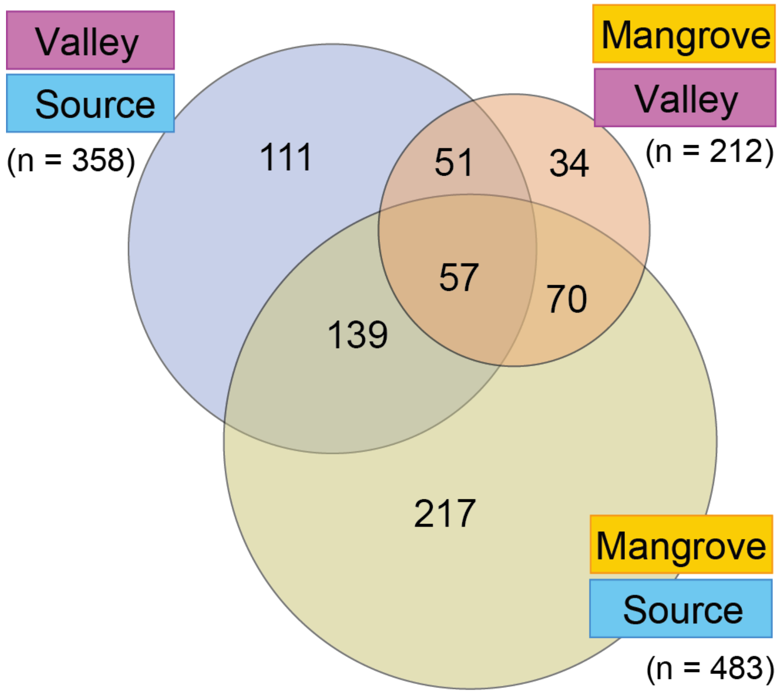

Supplemental Figure 6. Overlap in taxa with significantly different abundances between sites.

Taxa, resolved down to genus level, that are significantly different between pairwise comparisons of sites (ANCOM-BC, q-value <0.05) are shown as circles. Venn diagram overlap of these circles indicates taxa that are significantly different between two or three of these pairwise comparisons.

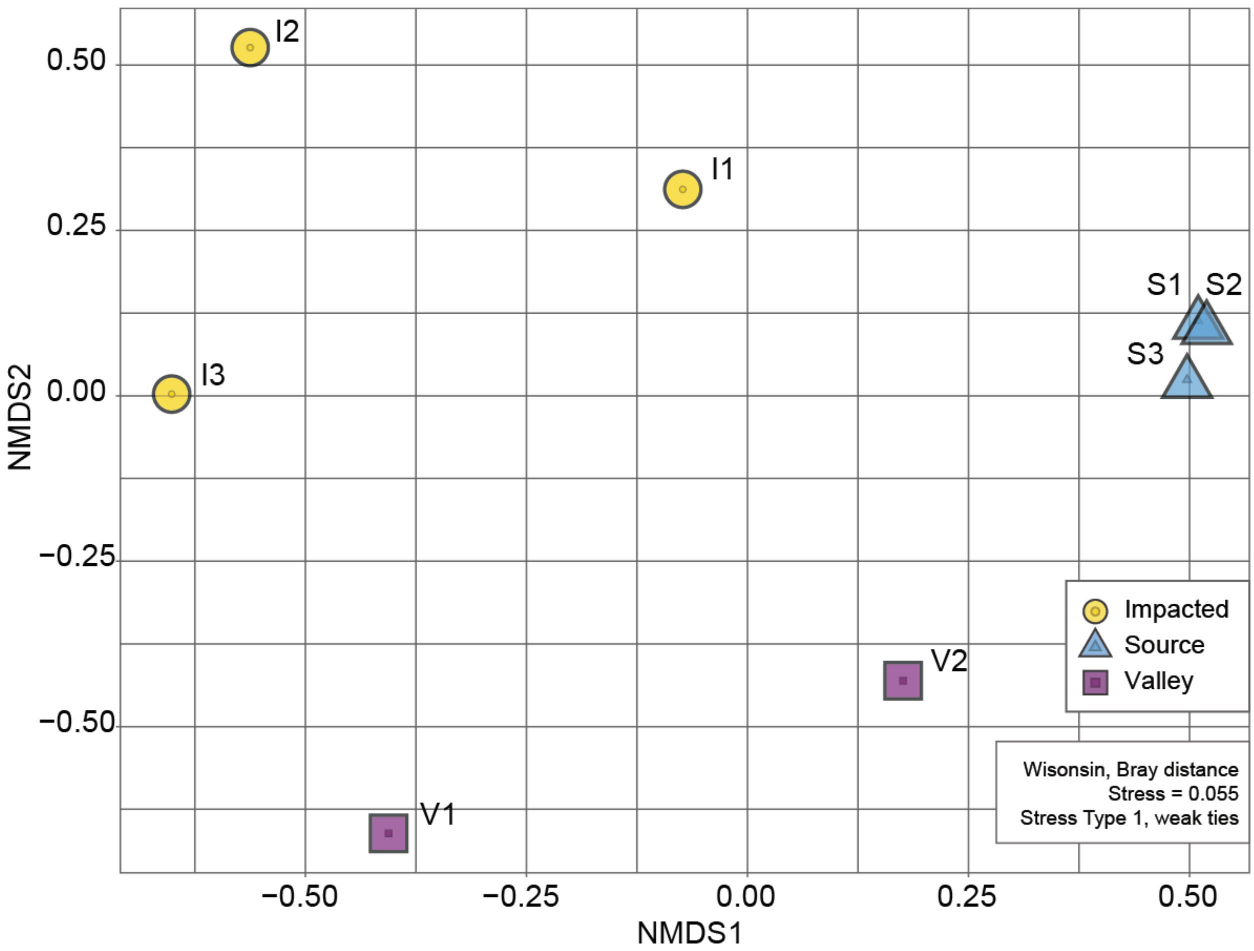

Supplemental Figure 7. NMDS plot

Supplemental Tables

Site	Sample-id	Temperature	D.O.	pH	Salinity
Source	S1	29.9	7.76	7.07	0
Source	S2	30.4	6.07	6.79	0
Source	S3	29.9	7.11	7.07	0
Valley	V2	30.9	9.35	7.26	0
Valley	V3	31	9.26	6.8	0
Impacted-Mangrove	I1	29.7	8.41	7.45	13.3
Impacted-Mangrove	I2	29.3	8.11	7.47	13.3
Impacted-Mangrove	I3	29	8.11	7.47	13.3

Sample-id	O. M.	Zn	Cu	Pb	Cr	Ni	Cd
S1	8.96	0.14	0.14	0.02	0.1	0.02	0
S2	8.75	0.14	0.14	0.02	0.1	0.02	0
S3	8.88	0.14	0.14	0.02	0.1	0.02	0
V2	6.67	0.07	0.02	0.01	0.1	0.01	0
V3	6.67	0.07	0.02	0.01	0.1	0.01	0
I1	9.93	0.63	0.13	0	0.13	5.17	0
I2	9.81	1.01	0.18	0.01	0.01	7.86	0
I3	9.9	0.63	0.18	0	0.16	7.86	0

Supplemental Table 1. Measured environmental variables

FWD: GTGCCAGCMGCCGCGGTAA
REV: GGACTACHVGGGTWTCTAAT

From: Caporaso, J. G., Lauber, C. L., Walters, W. A., Berg-Lyons, D., Lozupone, C. A., Turnbaugh, P. J., Noah Fierer, N., & Knight, R. (2011). Global patterns of 16S rRNA diversity at a depth of millions of sequences per sample. Proc Natl Acad Sci USA 108, 4516–4522. http://doi.org/10.1073/pnas.1000080107

Supplemental Table 2. Primers used in this study

Site	Sample_name	input	filtered	percentage passed filter
Source	I1	210701	195847	92.95
Source	I2	188197	172770	91.8
Source	I3	207034	192356	92.91
Impacted	S1	217495	202531	93.12
Impacted	S2	218935	203314	92.87
Impacted	S3	203580	188949	92.81
Valley	V1	117154	105178	89.78
Valley	V2	113141	97367	86.06

Sample_name	denoised	merged	percentage merged	Non-chimeric	Percentage non-chimeric
I1	185479	118785	56.38	108360	51.43
I2	160887	108244	57.52	103627	55.06
I3	182781	123340	59.57	113229	54.69
S1	194860	158272	72.77	148406	68.23
S2	196307	169337	77.35	162089	74.04
S3	181558	150083	73.72	142027	69.76
V1	97765	64048	54.67	60236	51.42
V2	87950	43068	38.07	41479	36.66

Supplemental Table 3. Denoising data quality control statistics. Reads were filtered, denoised, merged, and tested for chimera by Qiime2. All analysis in this study is based on the Non-chimeric reads.

Supplemental Table 4. ASV feature table.

Generated by QIIME2, supplemental file 1.

Supplemental Table 5. Taxa feature table.

Generated by QIIME2, supplemental file 1.

Supplemental Table 6. Genus level feature table.

Generated by QIIME2, supplemental file 1.

Supplemental Table 7. Stable genus.

Generated by Supplemental file 4. Collection of taxa, resolved down to the genus level, common between all sites. Taxa are required to have a relative abundance of at least 0.1% of reads per site, in at least 2 replicates per site, or at least 1% of reads in one replicate.

Supplemental Table 8. Singular genus

Generated by Supplemental file 5. Taxa are required to be minimally present at only one site. Minimal presence was defined as being greater than 0.001% of the total population per site, or being, on average, greater than 0.0001% of the population per site per replicate.

Supplemental Table 9. Results of ANCOM-BC between Spring and Valley.

Generated by QIIME2, supplemental file 1.

Supplemental Table 10. Results of ANCOM-BC between Valley and Mangrove.

Generated by QIIME2, supplemental file 1.

Supplemental Table 11. Results of ANCOM-BC between Mangrove and Spring.

Generated by QIIME2, supplemental file 1.

	I1	I2	I3	S1	S2	S3	V1
**I2**	0.3666529						
**I3**	0.35278	0.3785686					
**S1**	0.717329	0.693554	0.6753772				
**S2**	0.731515	0.699396	0.684452	0.2535798			
**S3**	0.724365	0.683713	0.61573	0.300265	0.3210206		
**V1**	0.693087	0.607335	0.476489	0.694608	0.704574	0.6173968	
**V2**	0.704052	0.522712	0.666436	0.757814	0.767188	0.744857	0.515989

Call:
metaMDS(comm = veganifyOTU(physeq), distance = distance)

global Multidimensional Scaling using monoMDS

Data: wisconsin(sqrt(veganifyOTU(physeq)))
Distance: bray

Dimensions: 2
Stress: 0.05564482
Stress type 1, weak ties
Best solution was repeated 4 times in 20 tries
The best solution was from try 15 (random start)
Scaling: centring, PC rotation, halfchange scaling
Species: expanded scores based on ‘wisconsin(sqrt(veganifyOTU(physeq)))’

Supplemental Table 12. Bray_distance_matrix.txt

***VECTORS

	PC1	PC2	r2	Pr(>r)
**Temperature**	0.86199	0.50693	0.5877	0.107
**D.O.**	0.98066	-0.1957	0.5975	0.118
**pH**	-0.27208	-0.96227	0.3293	0.356
**Salinity**	-0.20165	-0.97946	0.4082	0.292
**O.M.**	-0.88273	-0.46988	0.5704	0.122
**Zn**	-0.33603	-0.94185	0.6532	0.063
**Cu**	-0.95061	-0.31039	0.7979	0.011
**Pb**	-0.76224	0.64729	0.2925	0.451
**Cr**	0.27174	0.96237	0.3779	0.298
**Ni**	-0.34514	-0.93855	0.4413	0.23
**Cd**	0	0	0	1

Signif.	codes:	‘*’ 0.05	‘.’ 0.1	
Permutation:	free			
Number	of	permutations:	999	

Supplemental Table 13. PCA_results

Supplemental Files

Supplemental File 1. QIIME2 commands.

File name: Qiime_Project_Osun_6.sh

Contains DADA2, ASV to taxa assignment, taxonomic collapse to taxonomic levels, rarefaction, alpha-diversity, beta-diversity, ANCOM-BC.

Supplemental File 2. High relative abundance classes and phyla as barplots

File name: Make_stacked_bar_plots.py

Used in the generation of figure 1A

Supplemental File 3. Relative abundance heatmap plot.

File name: Heatmap.R

Used to generate Supplemental figure 4

Supplemental File 4. Stable genera identification and plotting.

File name: stable_genus.py

Used to generate Figure 1B, Supplemental Table 7.

Supplemental File 5. Singular genera identification and plotting.

File name: singular_genus.py

Used to generate Supplemental Figure 5, Supplemental Table 8

Supplemental File 6. ANCOM-BC plotting and plotting.

File name: volcano_plot_ancom.py

Used to generate Figure 3A, B, C.

Supplemental File 7. NMDS using Bray-Curtis

File name: Ordinate_nmds_bc.r

Used to generate Supplemental Figure 7, Supplemental Table 12

Supplemental File 8. PCA using Envfit

File name: envfit.r

Used to generate Figure 4B, Supplemental Table 13
